# MTI-101 treatment inducing activation of Stim1 and TRPC1 expression is a determinant of response in multiple myeloma

**DOI:** 10.1038/s41598-017-02713-0

**Published:** 2017-06-02

**Authors:** Michael F. Emmons, Nagaraju Anreddy, Javier Cuevas, Kayla Steinberger, Shengyu Yang, Mark McLaughlin, Ariosto Silva, Lori A. Hazlehurst

**Affiliations:** 1 Tumor Biology Department, Chemical Biology and Molecular Medicine Program, H. Lee Moffitt Cancer Center and Research Institute, Tampa, FL 33612 USA; 2 Modulation Therapeutics, Inc., 3802 Spectrum Boulevard, Suite 124, Tampa, FL 33620 USA; 3 Department of Pharmaceutical Science, University of West Virginia, Morgantown, WV 26506 USA; 4 Pharmacology and Physiology Department, University of South Florida, Tampa, FL 33620 USA; 5 Department of Cancer Imaging and Metabolism, H. Lee Moffitt Cancer Center and Research Institute, Tampa, FL 33612 USA

## Abstract

The emergence of drug resistance continues to be a major hurdle towards improving patient outcomes for the treatment of Multiple Myeloma. MTI-101 is a first-in-class peptidomimetic that binds a CD44/ITGA4 containing complex and triggers necrotic cell death in multiple myeloma cell lines. In this report, we show that acquisition of resistance to MTI-101 correlates with changes in expression of genes predicted to attenuate Ca^2+^ flux. Consistent with the acquired resistant genotype, MTI-101 treatment induces a rapid and robust increase in intracellular Ca^2+^ levels in the parental cells; a finding that was attenuated in the acquired drug resistant cell line. Mechanistically, we show that pharmacological inhibition of store operated channels or reduction in the expression of a component of the store operated Ca^2+^ channel, TRPC1 blocks MTI-101 induced cell death. Importantly, MTI-101 is more potent in specimens obtained from relapsed myeloma patients, suggesting that relapse may occur at a cost for increased sensitivity to Ca^2+^ overload mediated cell death. Finally, we demonstrate that MTI-101 is synergistic when combined with bortezomib, using both myeloma cell lines and primary myeloma patient specimens. Together, these data continue to support the development of this novel class of compounds for the treatment of relapsed myeloma.

## Introduction

Although there has been considerable progress in the treatment and survival rates of patients with multiple myeloma (MM), this malignancy remains an essentially incurable disease in dire need of new treatment strategies^1, 2^. We propose that targeting Ca^2+^ homeostasis is a tractable approach for treating MM that is resistant to standard-of-care agents. In support of this notion, recent studies have shown that cancer cells rewire their Ca^2+^ circuitry, including increased expression of components of store-operated channels (SOC) such as Ca^2+^ Release-activated Ca^2+^ Modulator 1 (Orai1), stromal interaction molecule 1 (STIM1), and the transient receptor potential channel 1 (TRPC1)^3, 4^. Moreover, SOCs appear to contribute to oncogene-mediated proliferation, migration and metastasis of cancer cells^5–7^. Accordingly, we reasoned that remodeling Ca^2+^ homeostasis of cancer cells provides an attractive therapeutic opportunity, as Ca^2+^ overload can trigger cell death^8^.

Intracellular Ca^2+^ levels are controlled by signals emanating from the plasma membrane, including G-protein-coupled receptors (GPCR), receptor tyrosine kinases (RTK), and cell adhesion molecules, including CD44^9^. Ca^2+^ homeostasis relies on the activation of specific phospholipases, including phospholipase-C β (PLCβ) by Gq/11 GPCRs or Phospholipase-C γ (PLCγ) by RTKs. These phospholipases cleave phosphatidylinositol 4,5-bisphosphate (PIP_2_) into the secondary messenger’s inositol triphosphate (IP3) and diacylglycerol (DAG). IP3 binds to the inositol triphosphate type 3 receptor (IP3R) on the endoplasmic reticulum (ER) membrane, which causes release of ER Ca^2+^ stores into the cytosol. ER Ca^2+^ depletion is then sensed by the scaffold protein STIM1, which changes its conformation and causes aggregation in the ER just below the cell membrane. Upon aggregation, STIM1 interacts with Orai1 and TRPC1, an essential components of SOC, and this interaction then promotes Ca^2+^ influx into cytosol^10, 11^.

A large body of data suggests that alterations in Ca^2+^ homeostasis can provoke necrosis. Under normal physiological conditions, extracellular Ca^2+^ is 5 mM whereas intracellular free Ca^2+^ ranges from 50 nM in the cytosol to ~500 µM in the ER. Specifically, prolonged elevation of free cytoplasmic Ca^2+^ (>1 µM) triggers mitochondria Ca^2+^ overload^12^, the opening of the mitochondrial permeability transition pore and the depletion of ATP, which leads to necrosis^13^. Furthermore, increased levels of cytoplasmic Ca^2+^ triggers the activation of Ca^2+^-dependent calpain proteases that permeabilize lysosomal membranes, thereby releasing lysosomal enzymes into the cytoplasm that also contribute to necrotic cell death^14^.

We recently showed that a D-amino acid linear peptide coined HYD1 and a more potent second-generation cyclized analog coined MTI-101 binds to a CD44/ITGA4-containing complex and provokes necrotic cell death^15–17^. The cell death pathway elicited by this novel class of molecules includes increased levels of reactive oxygen species (ROS), depolarization of the mitochondrial membrane potential, and depletion of ATP, all hallmarks of necrosis. Historically, necrosis was thought an uncontrolled form of cell death triggered by bioenergetic events that lead to a loss in osmolality, organelle and cell swelling and ultimately, cell lysis^18^. However, more recent studies have shown that necrosis can be triggered by “necroptotic” signaling pathways, including the Ripk1/Ripk3 circuit directed by tumor necrosis factor-alpha (TNFα)^19–21^.

Our recent studies demonstrated that MTI-101-induced cell death was only partially dependent on the TNFα-Ripk1/Ripk3 necroptotic pathway^16^. To gain insights into additional determinants of MTI-101-induced necrosis, we performed gene expression profiling on an acquired drug resistant cell line and found that genes predicted to attenuate store operated mediated Ca^2+^ flux were attenuated. Based on these data we hypothesized that Ca^+^ flux was a determinant of MTI-101 induced cell death in myeloma cell lines and primary patient specimens. To address our hypothesis we used both shRNA strategies and pharmacological approaches to attenuate store operated Ca^2+^ flux and showed that this pathway was indeed a determinant of MTI-101 induced cell death.

## Results

### Treatment with MTI-101 or HYD1 Increases Intracellular Ca^2+^ Levels in MM Cells

**Figure 1:**
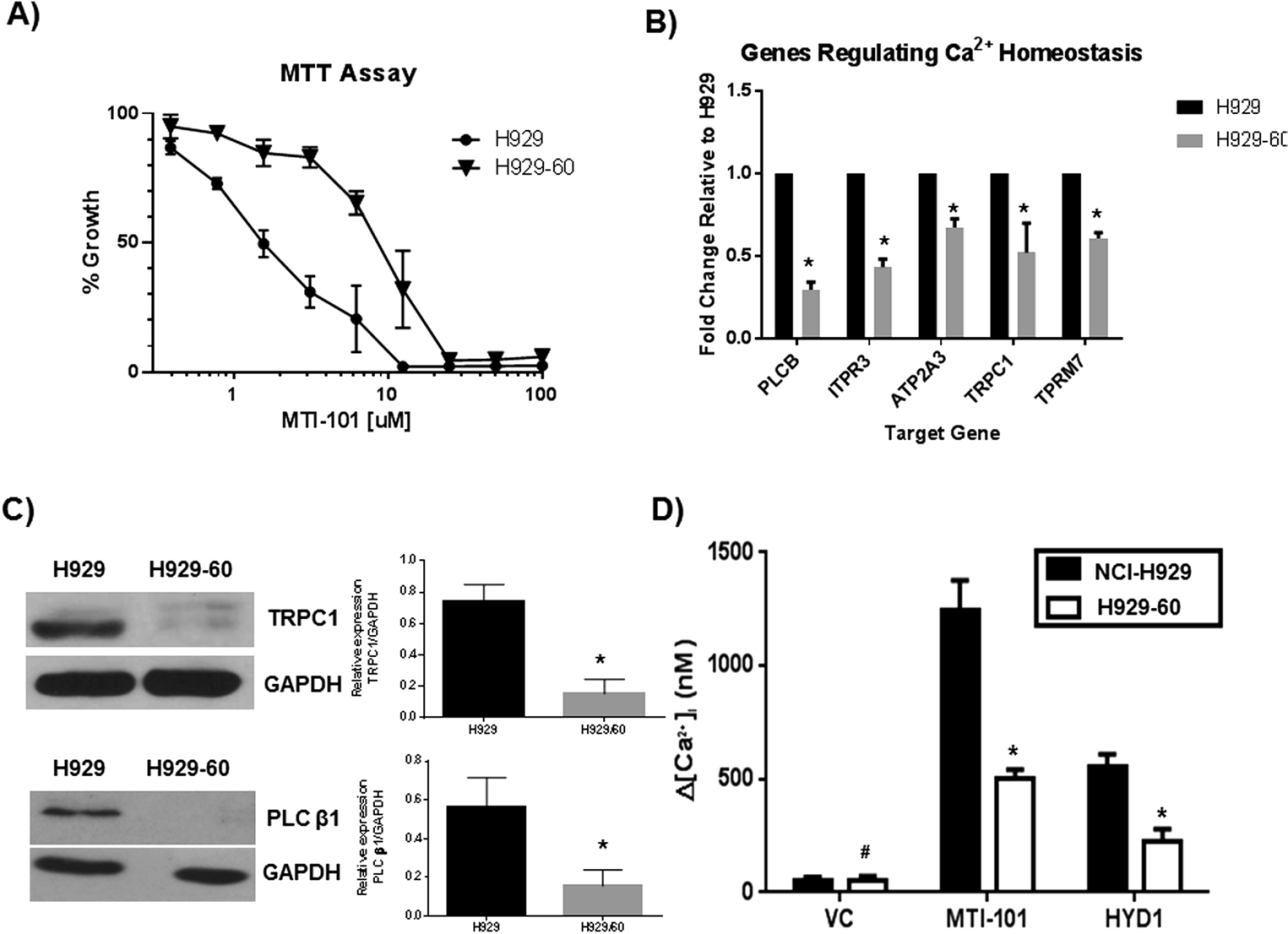
Selection for HYD1 resistance in NCI-H929 MM cells correlates with changes in Ca^2+^ signaling pathways. (**A**) Effect of MTI-101 on parentral (NCI-H929) and acquired drug resistant H929-60 cells. The H929-60 cells were developed by continuous exposure to the first generation linear peptide referred to as HYD-1. The H929-60 cell line is cross resistant to MTI-101 induced growth arrest. IC50 values of MTI-101 were determined using the MTT cell viability assay. Each value in the curve is the average of 3 independent experiments ± standard deviation. The resistant cell line was 7.8 fold resistant (n = 3 independent experiments p < 0.05 t-test). (**B,C**) Select genes were further validated by real time RT-PCR (n = 3 independent experiments p < 0.05, Student’s t-test, multiple testing corrected by Bonferroni-Dunn method) and western blot analysis (n = 3 independent experiments, shown is a representative blot). (**D**) Effect of MTI-101 on intracellular Ca^2+^ levels in NCI-H929 and H929-60 cells. H929 and H929-60 MM cells were adhered to cell-tak coated plates and were incubated with Fura-2 dye for 30 min. Time-lapse images were taken every 10 seconds for either 60 min after treatment with 75 µM HYD1 or 30 min after treatment with 3 µM MTI-101. Drug was delivered using a constant flow rate of continuous exposure of the drug. A one-way ANOVA test was used to determine significance (^#^p > 0.05; *p < 0.05; n = 30 or greater).

To determine the mechanism by which HYD1 and its cyclic analogue MTI-101 induces cell death in NCI-H929 cells, we developed the HYD1-resistant isogenic cell line H929–60^15, 16^. As shown in Fig. 1A the IC50 value for H929 is 1.2 +/− 1.15 uM while for, H929-60 cells the IC50 value was 9.3 +/− 1.08 uM towards MTI-101 induced growth arrest as measured by MTT assays (n = 3 independent experiments p < 0.05, t-test). Gene expression profiling of the parental and drug resistant variant was used to determine pathways that were altered as cells gained resistance to MTI-101. Specifically, the expression of *PLC-β*, the IP3 isoform 3 receptor, *TRPC1*, and *TRPM7* were all reduced in H929-60 cells (Table 1). Moreover ATP2A3 the SERCA channel was also downregulated in the drug resistant line (Table 1). Interestingly, recent studies indicate that TRPC1 and Orai1 expression regulate STIM1-dependent Ca^2+^ entry^22, 23^. Moreover, TRPM7 is a substrate of MLKL (mixed-lineage kinase domain-like protein), which is phosphorylated by Ripk3 following treatment with TNFα, linking Ca^2+^ entry with the necroptotic cell death pathway^24^. As shown in Fig. 1B a subset of the genes identified by microarray analysis were validated by real-time RT-PCR (n = 3 independent experiments performed in triplicates p < 0.05, Student’s t-test) and western blot analysis, respectively (n = 3 independent experiments). Interestingly, TRPM6 and TRPM8 were increased in the resistant cell line and may contribute to maintaining Ca^2+^ homeostasis in the resistant cell line despite reduced expression of the store Ca^2+^ operated channel pathway.

**Table 1:** Microarray gene expression profiling of NCI-H929 and H929-60 cells.

Gene Symbol	Average Signal +/− SD (n = 3) H929	Average Signal +/− SD (n = 3) H929-60	Fold Change H929-60/H929	p-value (paired t-test)
ITPR3	891 +/− 3.8	474 +/− 33	0.53	0.0027
PLCB1	525.5 +/− 126	325.6 +/− 57.4	0.66	0.045
TRPC1	241.7 +/− 45.8	138.5 +/− 20.24	0.54	0.024
TRPM7	328.8 +/− 31	208.4 +/− 13.4	0.63	0.013
ATP2A3	1215 +/− 139	557 +/− 75	0.46	0.008
TRPM6	20.4 +/− 5.4 (A)	146 +/− 15.9	7.2	0.008
TRPM8	65.6 +/− 31	228.4 +/− 42	3.5	0.018

The mean expression values of the three independent experiments were compared between NCI-H929 and H929-60 cell lines. Fold change was calculated by ratio between NCI-H929 H929-60 cell lines.

To test if HYD1 or MTI-101 treatment affected intracellular Ca^2+^ levels, NCI-H929 and H929-60 cells were loaded with fura-2 dye, and time-lapse images were taken every ten seconds for either 60 min (HYD1) or for 30 min (MTI-101) after treatment. Treatment with both HYD1 (75 µM) and MTI-101 (3 µM) triggered increases in intracellular Ca^2+^ levels in NCI-H929 and H929-60 cells. Furthermore, the levels of intracellular Ca^2+^ pools that were induced by MTI-101 were reduced in H929-60 *vs*. NCI-H929 cells (Fig. 1D), suggesting that HYD1- and MTI-101-induced MM death was due to Ca^2+^ overload. NCI-H929 cells were thus examined to assess if there were differences in total Ca^2+^ levels or differences in temporal Ca^2+^ levels after HYD1 or MTI-101 treatment. MTI-101 is more efficient in eliciting a Ca^2+^ response in MM cells than HYD1, consistent with the increased potency of MTI-101 *vs*. its linear counterpart on inducing cell death^16^. Collectively, these findings suggest that acquisition of resistance to this class of compounds is associated with changes in genes predicted to rewire Ca^2+^ homeostasis.

### MTI-101-induced Increases in Intracellular Ca^2+^ are Caused by Release of Ca^2+^ From ER Stores and Ca^2+^ Entry via Store-operated Channels

**Figure 2:**
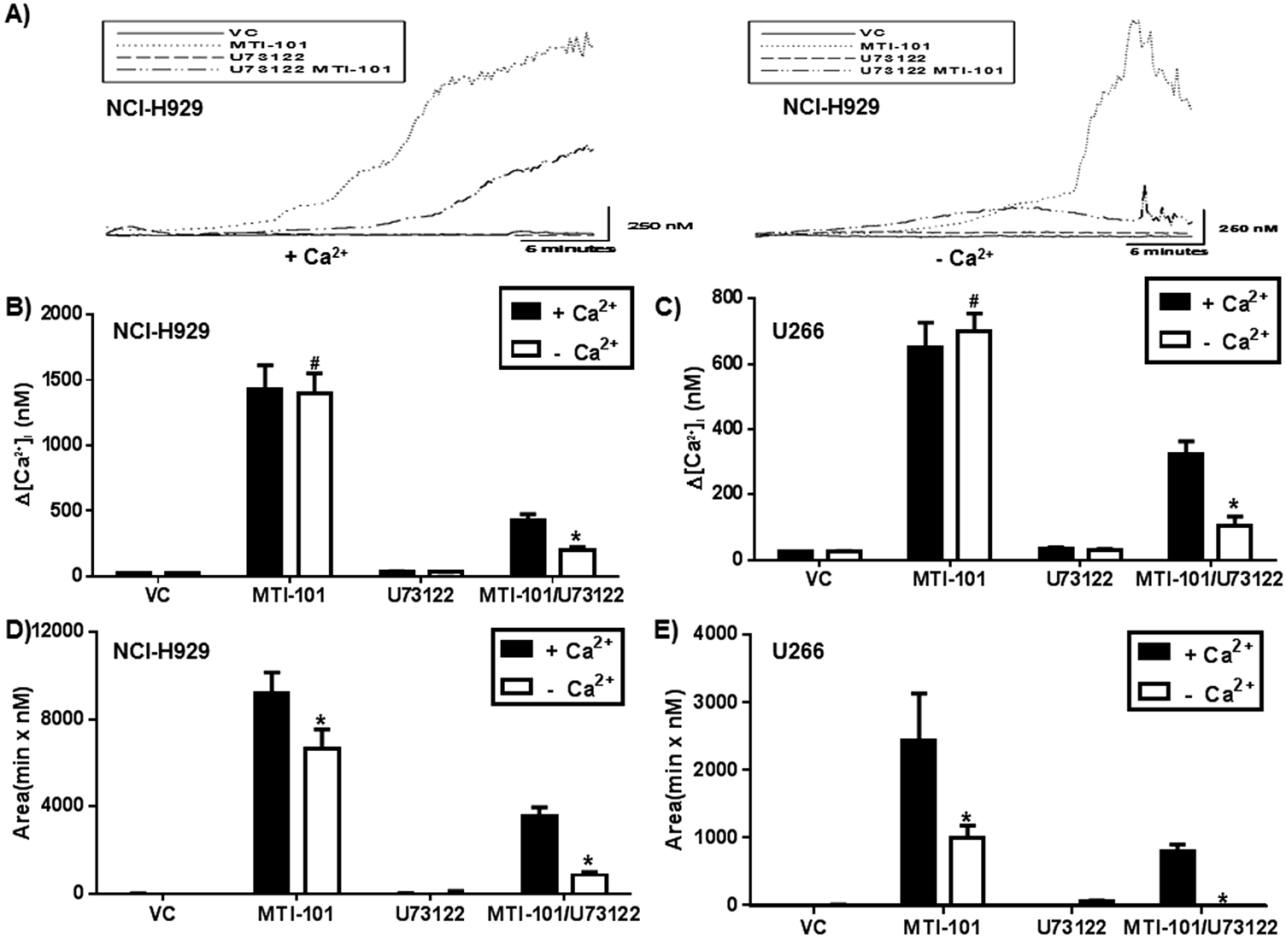
MTI-101 triggers release of ER Ca^2+^stores and influx of Ca^2+^ entry by plasma membrane store operated channels (SOC). (**A**) MTI-101 mediated intracellular Ca^2+^ levels were measured by using Fura-2 dye. NCI-H929 cells were adhered to cell-tak coated plates and then incubated with Fura-2 dye +/− 2 uM U73122 for 30 min in PSS containing Ca^2+^ and PSS without Ca^2+^. Time-lapse images were taken every 10 seconds for 30 min after treatment with 3 µM MTI-101. A representative H929 cell closest to the median Ca^2+^ response of the whole population is shown. The peaks were then measured and subtracted from the baseline Ca^2+^ levels. A representative experiment for (**B**) NCI-H929 and (**C**) U266 is shown. (**D** and **E**) The total level of Ca^2+^ induced by MTI-101 was determined by measuring the area under the curve for each individual cell. A one-way ANOVA test determined significance (^#^p > 0.05; *p < 0.05; n = 30 or greater). Shown is a representative experiment performed in triplicate.

Increase in intracellular Ca^2+^ occur either by influx through the plasma membrane channels or exchangers (*i.e*., voltage-gated channels, SOC, Na^+^-Ca^2+^ exchanger) or via release from internal stores such as the ER^25–27^. One means of Ca^2+^ release from ER stores is through the opening of the IP3R caused by PLC activation^25^. To test if activation of PLC was involved in MTI-101-induced intracellular Ca^2+^ increases, NCI-H929 or U266 cells were pre-treated with 2 µM or 1 µM of the PLC inhibitor U73122^28^, respectively, and were then treated with MTI-101. This assay was performed in the presence and absence of extracellular Ca^2+^ to determine the effects of MTI-101 on Ca^2+^ influx to cells. After treatment with MTI-101, the effects on Ca^2+^ levels were measured using Fura-2. U73122 treatment decreased MTI-101-induced Ca^2+^ levels in both NCI-H929 (Fig. 2A) and U266 cells (data not shown); a finding that was further attenuated upon removal of extracellular Ca^2+^ indicating that increased levels of Ca^2+^ induced by MTI-101 occur via both release of ER stores and Ca^2+^ entry via store-operated channels. Although, we could not detect a decrease in peak levels of Ca^2+^ in the absence of the PLC inhibitor (Fig. 2B,C), removing extracellular Ca^+2^ reduced the MTI-101-induced sustained increases in Ca^2+^ levels (as measured by the area under the curve) in both H929 and U266 cells (Fig. 2D,E). Together these data indicate that Ca^2+^ release from the ER, via activation of the IP3R, contributes to the peak levels and Ca^2+^ entry through plasma membrane channels contribute to the sustained high levels of Ca^2+^ observed following treatment with MTI-101.

**Figure 3:**
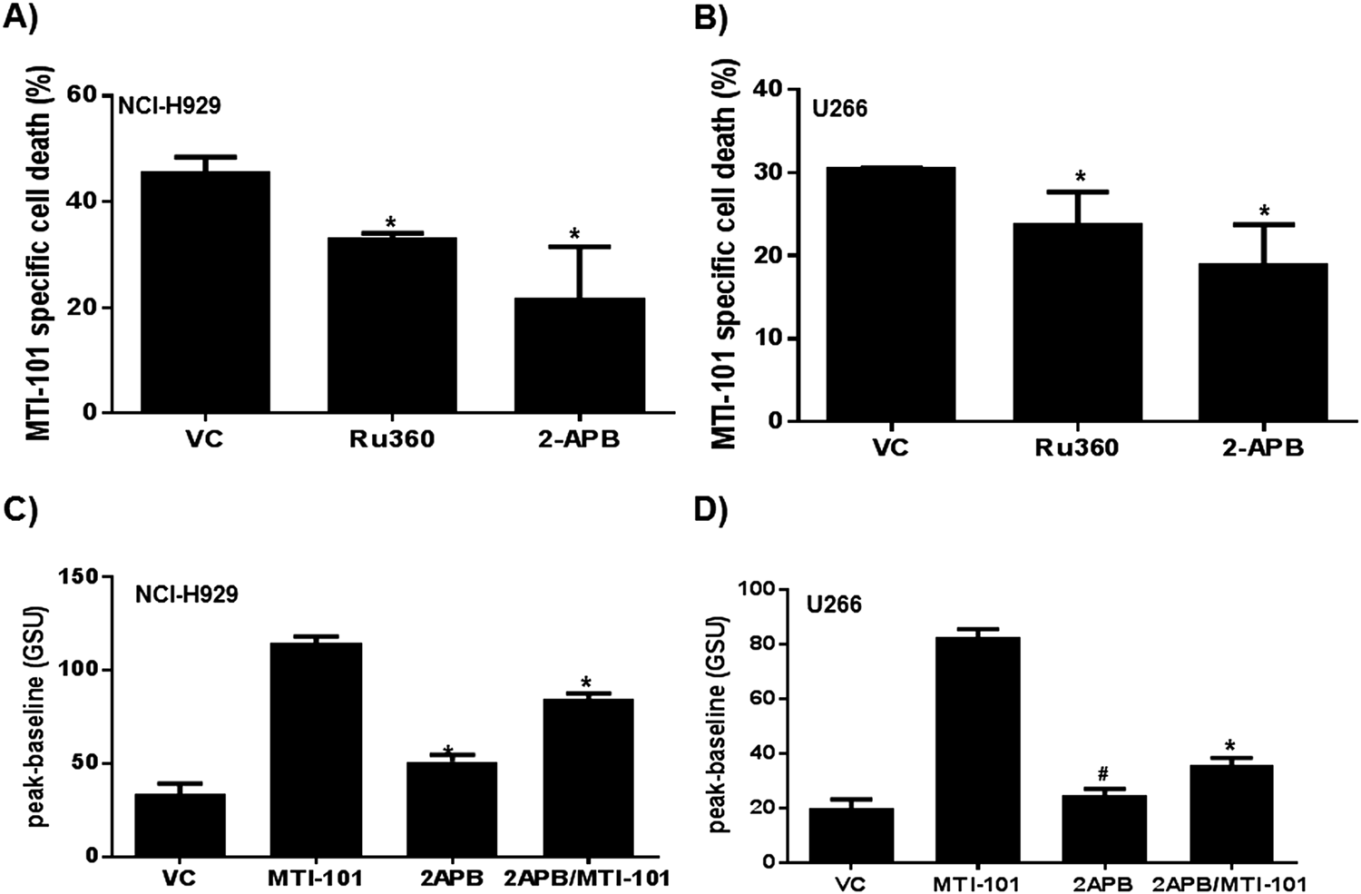
Blocking Ca^2+^ store operated calcium channels or inhibiting Ca^2+^ entry into mitochondria impairs MTI-101 induced cell death. (**A**) NCI-H929 and (**B**) U266 MM cells were pretreated with 50 μM-APB and 5 μM Ru360 for 30 min or vehicle control (VC). After 30 min cells were treated with 10 or 20 μM of MTI-101, respectively, for 2 hr. MTI-101 specific cell death was measured by FACS analysis. Shown is a representative experiment performed in triplicate. (**C**) NCI-H929 and (**D**) U266 MM cells were pretreated with 50 µM of 2-APB followed by treatment with 10 or 20 µM of MTI-101, respectively. Ca^2+^ levels were measured by fluo4 AM. The Y-axis is depicted as gray scale units (GSU or pixel intensity).

These findings suggest that blocking Ca^2+^ release from ER stores and/or Ca^2+^ entry would inhibit MTI-101-induced cell death. Thus, we tested whether 2-APB, which blocks Ca^2+^ release through the IP3R as well as SOC-mediated Ca^2+^ entry^29^, affected MTI-101-induced myeloma cell death. Notably, both NCI-H929 and U266 cells pretreated with 2-APB are resistant to MTI-101-induced cell death (Fig. 3A,B). To determine whether Ca^2+^ entry into mitochondria contributes to death we blocked the mitochondria uniporter with RU360. As shown in Fig. 3A,B pretreatment with RU360 blocks MTI-101 induced cell death (p < 0.05, students T-test). These data suggest that MTI-101-induced cell death is, at least in part, due to Ca^2+^ overload of mitochondria. As shown in Fig. 3C,D, cells pre-treated with 2-APB and then treated with MTI-101 inhibits MTI-101-mediated increases in intracellular Ca^2+^ (Fig. 3C,D). Thus, blocking increases in intracellular Ca^2+^ is sufficient to inhibit MTI-101 induced cell death.

**Figure 4:**
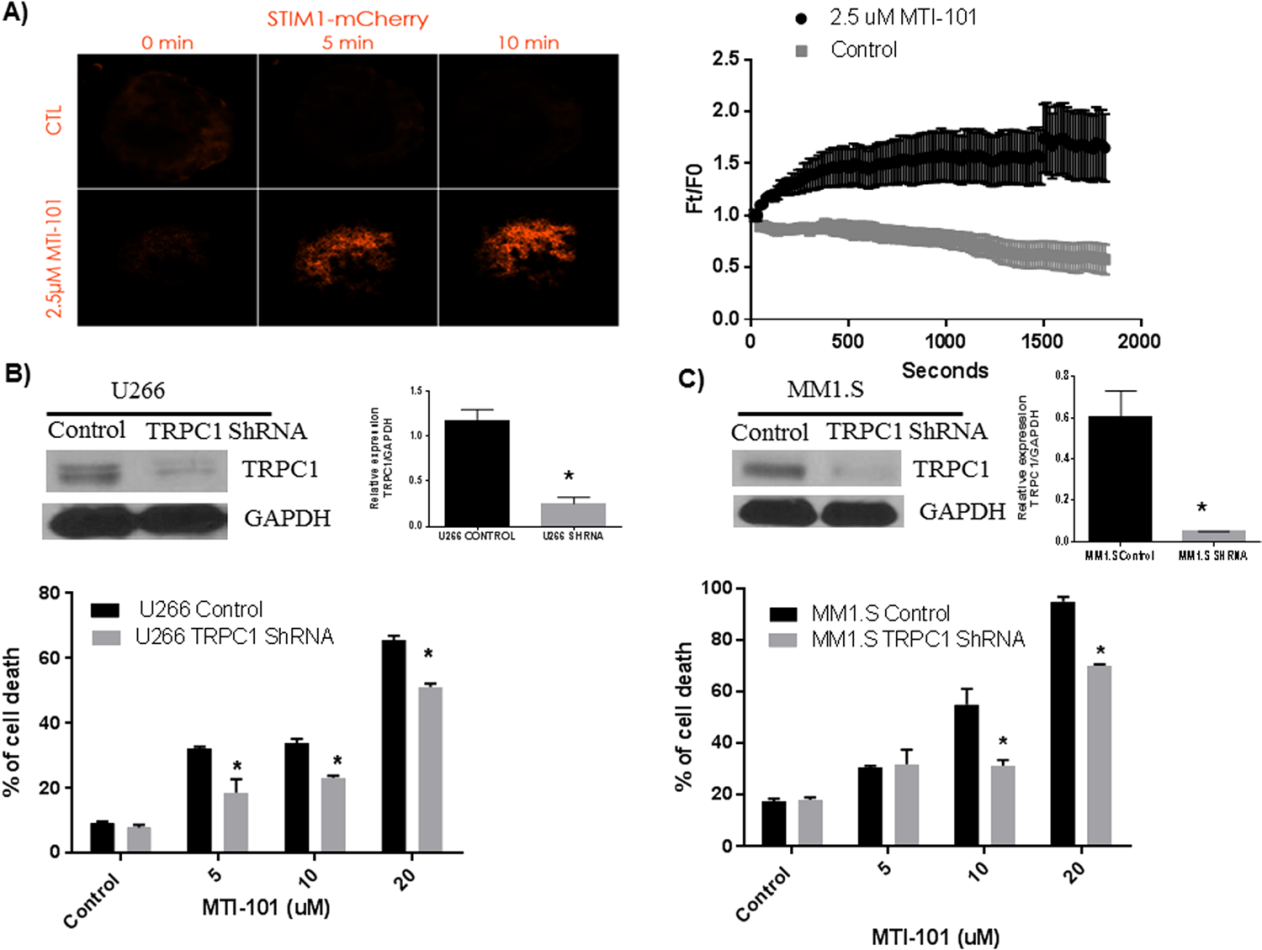
MTI-101 activates STIM1 and reducing the expression of TRPC1 inhibits MTI-101 induced cell death. (**A**) U226 cells over expressing Stim1 m-cherry show increased trafficking to the plasma membrane following treatment with MTI-101 compared to control cells. Using TIRF microscopy, images of individual cells (n = 7) at each time point were visualized, analyzed, and linked through time. TIRF images were collected every 15 seconds over 30 minutes with the experimental group receiving a single 2.5 μM treatment of MTI-101 at 30 seconds. Ft/F0 is the pixel intensity at the indicated time as denoted on the X axis divided by the average pixel density prior to drug treatment. Mean intensity data for STIM1 mCherry at each time point were extracted for comparisons. A representative cell from the control group and treatment group is shown before treatment (0 min) and after treatment (5 and 10 min). Shown is the mean and standard error of 7 cells for a representative experiment (p < 0.05, Two Way ANOVA). The experiment was repeated 3 independent times and similar data was obtained. (**B** and **C**). TRPC1 expression was reduced by using retroviral TRPC1 shRNA construct and expression was determined by Western Blot in U266 and MM1.S cells respectively. (**B** and **C See Supplemntal Data** S1). MM1.S and U266 cells were treated with different concentrations (5, 10 and 20 μM) of MTI-101 for 48 hr and cell death was measured using PI staining. Cells with reduced TRPC1 levels cells showed a significant reduction in MTI-101 induced cell death (p < 0.05, Student’s t-test).

To test whether MTI-101 induces activation of STIM1 as determined by the formation of punctae and trafficking to ER/PM junctions, we used TIRF microscopy, which images 100 nm proximity of the surface of the slide. As shown in Fig. 4A,B, U266 cells which overexpress STIM1 m-cherry have significantly increased levels of Stim1 as determined by total fluorescence intensity near the plasma membrane (p < 0.05, Two-way ANOVA). To test the specific role of TRPC1, a component of the store-operated channels we used shRNA to reduce the expression of TRPC1. As shown in Fig. 4C,D, reducing the expression of TRPC1 significantly inhibits MTI-101 induced cell death (p < 0.05, student’s t-test).

### MTI-101/Bortezomib Combination demonstrates Increased Anti-Myeloma Activity *in vitro* and *In Vivo*

**Figure 5:**
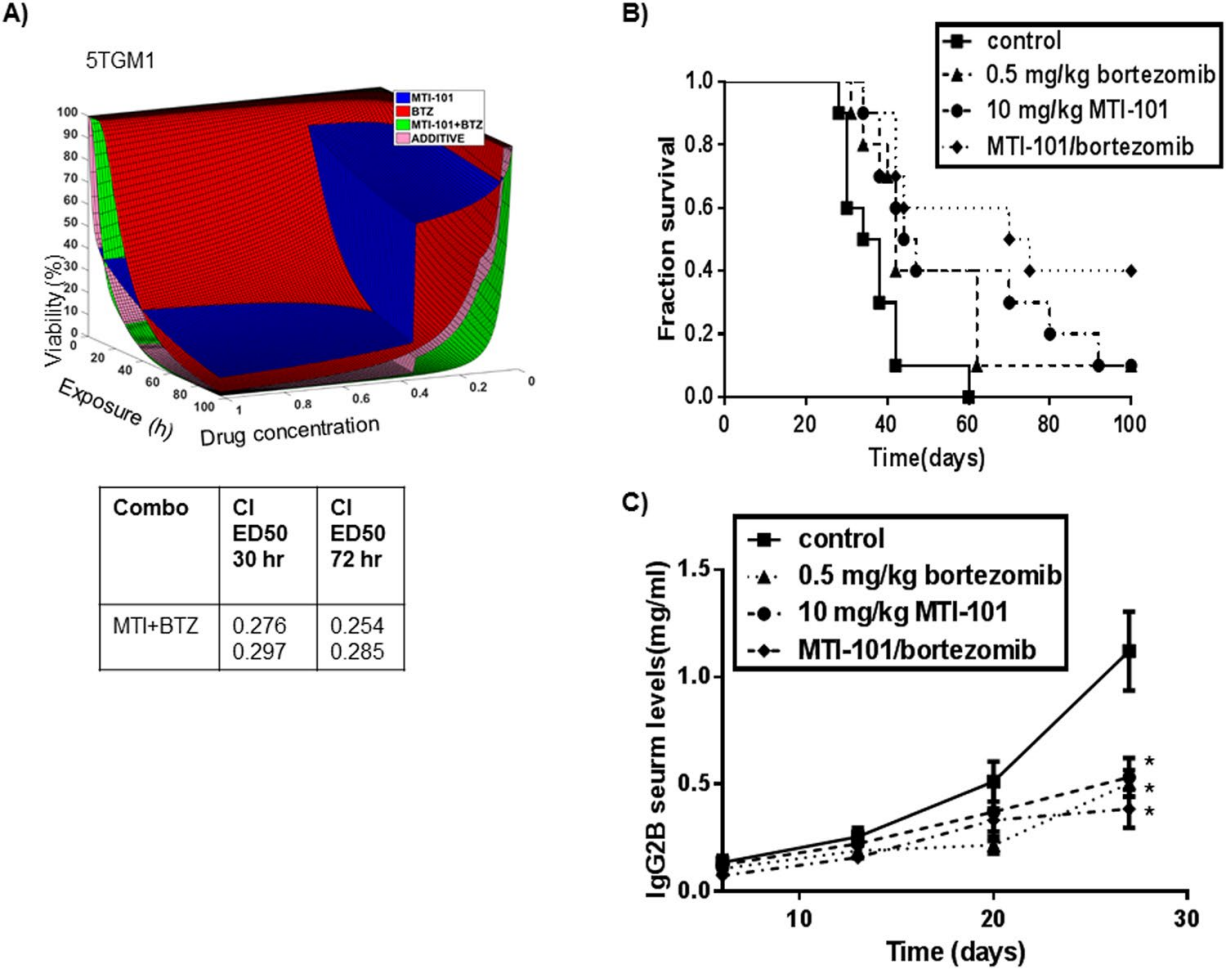
Combination treatment with MTI-101 and bortezomib augments survival *in vitro* and *in vivo*. (**A**) 5TGM1 cells were treated with varying concentrations of MTI-101, bortezomib or the combination of both drugs for 72 hours using a bone marrow stroma model as described in materials and methods. Combination indexes (CI) for duplicate wells were calculated at 30 and 72 hrs. (**B**) 1 × 10^6^ 5TGM1 MM cells were injected into 6–8 week old C57BL/KaLwRijHsd mice via tail vein. At day 10, mice were treated with 10 mg/kg of MTI-101 and/or 0.5 mg/kg bortezomib three times a week for three weeks (10 mice per group). Mice were monitored daily for survival. Mean survival times for vehicle control, bortezomib only (*p* < 0.02, log-rank sum), MTI-101 only (*p* < 0.01, log rank sum), and MTI-101/bortezomib (*p* < 0.001, log rank sum) combination therapy were 36, 42, 45.5, and 72.5 days, respectively. (**C**) IgG2B serum levels were measured by ELISA once a week for 4 weeks to determine myeloma levels in the peripheral blood. At day 28 all drug treatment groups showed a significant decrease in IgG2B levels compared to control animals (p < 0.05).

Several reports indicated that blocking L-type Ca^2+^ channels or blocking Ca^2+^ uptake by inhibiting the mitochondria uniporter blocks cell death induced by bortezomib. In contrast, 2-APB was not shown to block bortezomib-induced cell death. It is attractive to speculate that increased Ca^2+^ via L-type, and store-operated channels converge to cause synergistic cell death via Ca^2+^ overload of the mitochondria in myeloma cells^30, 31^. Thus we next sought to determine the activity of MTI-101 in combination with the standard of care agent bortezomib in 5TGM1 cells (Fig. 5A). For 5TGM1 cells we utilized a bone marrow stroma co-culture model and time lapse imaging of individual cells to determine viability as function of time. Combination index was calculated at select time points using the Calcusyn software^32, 33^. As shown in Fig. 5A, in 5TGM1 cells when MTI1-101 is combined with bortezomib synergy is observed, as defined by a combination index of less than 1 cells as early as 30 hours and was maintained to the endpoint of 72 hours. To test whether MTI-101 in combination with Bortezomib has anti-tumor activity *in vivo* C57BL/KaLwRijHsd mice (6- to 8-week old) were injected *i.v*. with syngeneic 5TGM1 MM cells and were then treated with MTI-101 (10 mg/kg) and/or bortezomib (0.5 mg/kg). As predicted, mice treated with MTI-101 or bortezomib had improved survival versus vehicle treated mice (Fig. 5B). Notably, the MTI-101/bortezomib combination had superior anti-myeloma activity versus single-agent treatment. Specifically, mean survival times for vehicle control, bortezomib only, MTI-101 only, and MTI-101/bortezomib combination therapy were 36, 42, 45.5, and 72.5 days, respectively (p < 0.05, Log-Rank Test) (Fig. 5B). To assess tumor burden in transplanted mice, IgG2B serum levels were determined (Fig. 5C). Interestingly we could not detect decreases in IgG2B levels with the combination at 4 weeks, which may reflect decreased fitness for cells that survive the combination treatment. We had previously reported that selection for resistance to MTI-101 resulted in a compromised CAM-DR phenotype and decreased adhesion to fibronectin and stroma cells^15^.

### Sensitivity to MTI-101 is augmented in Relapsed vs. Primary Multiple Myeloma

**Figure 6:**
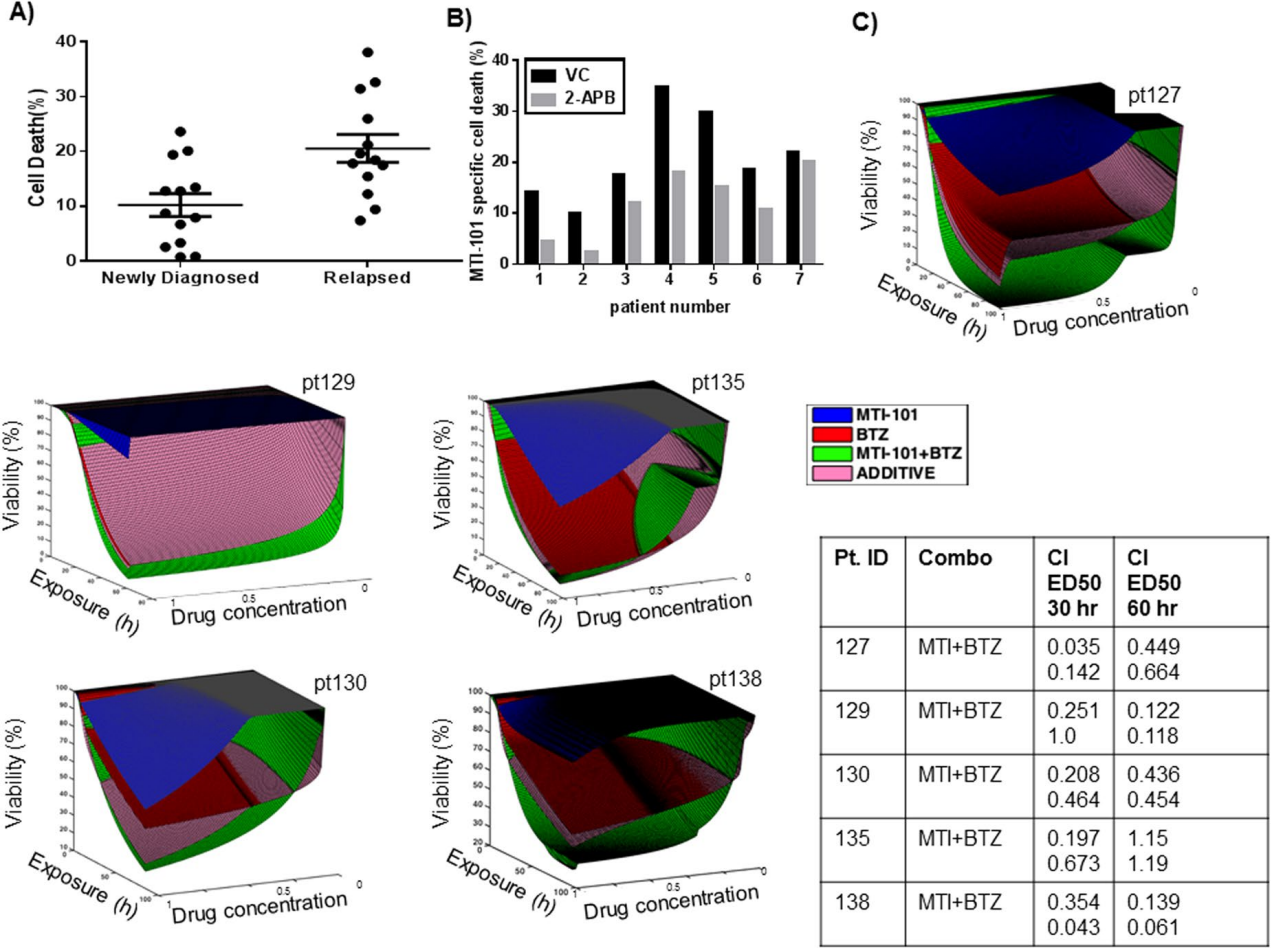
Relapsed myelomas are more sensitive to MTI-101. Specimens were separated into two groups depending on clinical diagnosis; either newly diagnosed or relapsed patients. (**A**) CD138+ cells were treated with 10 µM of MTI-101 for 24 hr. After 24 hr cell death was measured by Annexin V staining and FACS analysis. CD138 cells derived from patients which have relapsed on therapy were significantly more sensitive to MTI-101 induced cell death compared to CD138 cells obtained from newly diagnosed patients (p < 0.05, Student’s t-test) (**B**) CD138+ cells were pretreated with 50 µM 2APB or VC (vehicle control) for 30 min and then were treated with 10 µM MTI-101 for 24 hrs followed by FACS analysis. (**C**) Dose response surfaces for primary MM cells from patients treated *ex vivo* with bortezomib (50–0.6 nM, 3 fold serial dilution), MTI-101 (20–0.25 uM, 3 fold serial dilultions)) and combination. Exposure time was 96 hrs except for patient pt129 (60 hrs). The actual combination of MTI-101 and bortezomib (MTI-101 + BTZ) is more effective (higher kill) than theoretical additive effect (ADDITIVE) indicating synergy between two drugs. Combination indexes (CI) were calculated using CalcuSyn software at 30 and 60 hrs. Samples were performed in duplicate and independent combination indexes were calculated for each patient specimen.

To test the sensitivity of primary vs. relapsed myeloma specimens to MTI-101, CD138-positive malignant plasma cell fractions from thirteen newly diagnosed and twelve relapsed primary MM specimens were treated with MTI-101. Consistent with our previous studies using the HYD1 linear analog of MTI-101^15^, MTI-101 was significantly more potent (p < 0.05, Student’s *t*-test) in relapsed than newly diagnosed MM specimens (Fig. 6A).

As 2-APB pretreatment inhibits MTI-101-induced death of MM cell lines, we tested whether this Ca^2+^ blocker would also impair MTI-101-induced cell death of primary MM specimens. Indeed, patient samples pretreated with 50 µM 2-APB were also more resistant to MTI-101 treatment (Fig. 6B p < 0.05, Student’s t-test). Thus, similar to MM cell lines, primary MM cells treated with MTI-101 die, at least in part, due to Ca^2+^ overload. Finally, as shown in Fig. 6C, the synergy between MTI-101 and bortezomib was observed in 5 out of the 6 primary patients, with pt135 showing synergy at 30 but not 60 hrs. Together these data indicate that combining MTI-101 with bortezomib maybe a good strategy for the treatment of multiple myeloma.

## Discussion

MM is a disease that initially responds to chemotherapy. However, despite the range of mechanistically distinct therapies, the disease will eventually relapse and become refractory to further treatment, underscoring the need for novel treatment strategies. MM has high basal levels of ER stress due to the load of IgG production and is thus prone to initiate an unfolded protein response^34^. This increased ER load appears to contribute to the sensitivity of MM towards treatment with proteasome inhibitors. Our data support the notion that MM cells also rewire their Ca^2+^ circuitry, and that targeting Ca^2+^ signaling is an attractive therapeutic strategy for drug-resistant MM. Indeed, this may be a general feature of cancer cells, as demonstrated by increased expression of SOC and STIM1 in multiple tumor types^3, 4^ and the required roles for elevated Ca^2+^ pools in cancer cell migration, invasion and metastasis^5, 7^. Our findings suggest that remodeling the Ca^2+^ circuitry occurs at a cost to fitness, rendering cells vulnerable to death induced by Ca^2+^ overload.

Our findings indicate that the acquisition of resistance to HYD1 and MTI-101 is associated with remodeling the circuitry that controls Ca^2+^ homeostasis. Specifically, drug resistance correlated with the suppression of IP3R, PLC, TRPC1, and TRPM7 expression and increased expression of TRPM6 and TRPM8 (Table 1). This switch in expression might reflect a compensatory response allowing for similar basal levels of Ca^2+^ to support growth in the face of MTI-101 treatment. Moreover, in this study, we showed that MTI-101 treatment as a single agent induces increased intracellular Ca^2+^ levels; a finding that contributes to MTI-101 induced cell death in MM cells. Evidence to support the role of Ca^2+^ release from the ER included data showing that the PLC inhibitor partially blocks MTI-101 induced Ca^2+^ levels. In addition, we showed that the SOC channel and IP3 receptor inhibitor 2-APB attenuates MTI-101 mediated cell death in MM cell lines as well as primary patient derived CD138 plasma cells. Finally, we showed that MTI-101 induces STIM1 punctae near the plasma membrane, an event required for activation of store operated channels. Selection for resistance towards MTI-101 correlated with reduced expression of TRPC1 a component of the store operated channel. This finding guided our focus on the role of TRPC1. Indeed, we showed that a reduction TRPC1 partially inhibits MTI-101 induced MM cell death. Together, our results suggest that MTI-101 mediated increase in Ca^2^ is caused by release of Ca^2+^ from ER stores and Ca^2+^ entry via store-operated channel and that TRPC1 expression is a determinant of response in MM.

Treatment of cancer is typically most effective when combination strategies are implemented. Thus, it is important to place novel treatment strategies in context with standard of care agents. In this study, we showed that treatment with MTI-101 in combination with the standard of care agent Bortezomib demonstrates increased anti-myeloma activity *in vitro* and *in vivo*. Importantly, the synergy was maintained using CD138 cells derived from patients and cultured in an organotypic model which contained bone marrow stroma cell and collagen. These data indicate that MTI-101 in combination with the proteasome inhibitors is a tractable strategy for the development of this class of inhibitors. Mechanism of action of bortezomib includes inhibition of NF-κB activity and the induction of the unfolded protein response (UPR)^34, 35^. Accordingly, alterations in NF-κB pathways, the UPR and the induction of autophagy have been implicated in mediating resistance to bortezomib^36, 37^. Further, acquired resistance to bortezomib in cell line cultures has been linked to the identification of missense mutations (Ala49Thr) in the β5-subunit (PSMB5) of the proteasome and to increased expression of PSMB5^38, 39^. However, sequencing efforts have failed to identify mutations in *PSMB5* in clinical resistance to bortezomib^40^. Several reports indicated that blocking L-type Ca^2+^ channels or blocking Ca^2+^ uptake by inhibiting the mitochondria uniporter blocks cell death induced by bortezomib^30, 31^. It is attractive to speculate that synergistic cell death between MTI-101 and Bortezomib is driven via Ca^2+^ overload of the mitochondria in myeloma cells. However, recent studies indicate that inhibition of the proteasome can lead to necroptosis in a RIPK3 dependent pathway^41^. Thus, it is also feasible that the observed synergy between MTI-101 and bortezomib is due to augmentation of activation of RIPK3 leading to increased phosphorylation of MLKL and necroptosis. We currently favor the hypothesis that that as primary samples become resistant to bortezomib, myeloma cells may rewire Ca^2+^ homeostasis to favor the induction of Ca^2+^ flux via store-operated channels rather than L-type channels, which underlies increased sensitivity to MTI-101 induced cell death in specimens obtained from patients relapsing on therapy. More studies are required to fully understand the mechanistic underpinning of synergy when MTI-101 is combined with bortezomib and the increased activity of MTI-101 in relapsed patient specimens. In summary, MTI-101 remains an attractive novel class of compounds to test as a front line combination strategy with proteasome inhibitors or in the setting of proteasome inhibitor refractory disease.

## Materials and Methods

### Cells and Reagents

NCI-H929 and U266 multiple myeloma (MM) cells were obtained from American Type Culture Collection (Manassas, VA) and maintained in RPMI-1640 medium supplemented with 10% FBS and 1% penicillin/streptomycin/glutamine. NCI-H929 cells were also supplemented with 0.05 mM beta-mercaptoethanol. The HYD1 drug-resistant NCI-H929 cell line H929-60 was developed as previously described^15^. The parental and drug resistant line was validated by short tandem repeat (STR) analysis. 5TGM1 myeloma cells were derived from murine myeloma 5T33 and kept in similar media as U266 cells. All cell lines were tested for mycoplasma every six months. HYD1 (kikmviswkg) and MTI-101 were synthesized by Bachem (San Diego, CA). Bortezomib was purchased from Selleck Chem. The compounds U73122 (Sigma), 2-APB (Sigma) and Ru360 (Merck Millipore) were all used to inhibit Ca^2+^ signaling.

### Gene Expression Profiling

Gene expression profiling was performed as previously described^42^. Briefly, 100 ng of total RNA was isolated using a RNeasy mini kit (Qiagen). The oligonucleotide probe arrays used were the Human Genome U133 plus 2.0 Arrays. Data were processed and normalized using IRON. The mean expression values of the three experiments were compared between NCI-H929 and H929-60 MM and fold change were compared between mean expression in the two groups.

### Real-Time PCR Analysis

1 μg total RNA was used as a template to synthesize cDNA with the High Capacity cDNA Reverse Transcription Kit (Applied Biosystems, Foster City, CA; Cat. no. 4368814) with a reaction volume of 50 μL. Real-time PCR was performed on the ABI 7900HT Fast Real-Time PCR System (Applied Biosystems) using assays specific for each gene of interest. Each reaction well contained 5μL of Power SYBR Green PCR Master Mix (cDNA equivalent to 20 ng of total RNA and 400 nM each of forward and reverse amplification primers in a reaction volume of 10 μL. Cycling conditions were as follows: 95 **°**C for 10 minutes for polymerase activation, followed by 40 cycles of 95 **°**C for 15 seconds and 60 **°**C for 1 minute. Data analysis was performed using Sequence Detection System software from Applied Biosystems, version 2.4. The experimental Ct (cycle threshold) was calibrated against the endogenous control product GAPDH. Real Time RTPCR was performed by ARQ genetics LLC Bastrop, TX. The following primer sets were used for amplification.

ATP2A3 Forward primer CCGGAACCACATGCACGAAG; Reverse primer GGGGATGGCCATTCTGACCTC

PLCB1 (span Exon 4–5) Forward primer; GTGTCCGACAGCCTCAAGAA; Reverse primer ATCCCTGAGGGTCAGTCCTC

TRPC1 Forward primer CGGTTGTCAGAAACTAATGGAACG; Reverse primer CCTGAATTCCACCTCCACAAGA

TRPM7 Forward primer CTGCTTTTGATCTCCTGTCCTGT; Reverse primer CAGACAGCCCATATTGCCCT

ITPR3 Forward primer GTTCCTGACGTGTGACGAGT Reverse primer GCCACCAGGCAGTACTTGAT

HGAPDH-forward primer TGCCCTCAACGACCACTTTG; reverse primer CTCTTCCTCTTGTGCTCTTGCTG

### TIRF imaging of STIM1 mCherry

Imaging of STIM1 mCherry overexpressing U266 cells was performed using a total internal reflection fluorescence (TIRF) imaging system with objective lens 60x/1.49 Apo TIRF DIC in oil immersion using a Nikon Eclipse TE2000-E microscope (Melville, NY). Data were collected with a CoolSNAP HQ monochrome CCD camera (Tucson, AZ) and analyzed by NIS-Elements Software (Nikon Instruments, Melville, NY). mCherry-tagged STIM1 was excited with a 561-nm TIRF laser respectively, and emissions were collected using a 300-ms exposure time. TIRF images were collected every 15 seconds over 30 minutes with the experimental group receiving a single 2.5 μM treatment of MTI-101 at 30 seconds. Images of individual cells (n = 8) at each time point were analyzed and linked through time. Mean intensity data for STIM1 mCherry at each time point were extracted for comparisons. All experiments were carried out at 37 °C and 5% CO_2_.

### shRNA knockdown of TRPC1 protein

pVSV-G envelope vector was transfected into gag-pol packaging genes containing GP-293 cells (63150, Clontech Laboratories, Inc, CA) with TRPC1 shRNA construct, plasmids containing these sequences (TG308629c, Origene Technologies, MD) to produce infectious ecotropic retrovirus. The viral supernatants were collected 48 hours after transfection and used to infect cells in the presence of 4 μg/mL Polybrene (Sigma-Aldrich, St Louis, MO). Briefly, MM1.S and U266 cells were infected with TRPC1, Scrambled virus for 24 hr and then removed the virus and incubated the cells with fresh medium for 72 hours before selecting cells with Puromycin. TRPC1 expression was determined by Western blot with the TRPC1 antibody (c-133076, Santa Cruz Biotechnology, CA).

### Measurement of Intracellular Ca^2+^Concentrations

Intracellular free Ca^2+^ was measured using the Ca^2+^-sensitive dyes fura-2 and fluo-4 (Life Technologies). Glass-bottom microwell dishes (35 mm; Mattek Cultureware, Ashland, MA) were plated with 10-μL Cell-tak (BD Biosciences) per manufacturer’s instructions. Fura-2 and fluo-4 loading was performed by incubating the plated cells for 30 min at room temperature in either physiological saline solution (PSS). PSS is consist of 140 mM NaCl, 3 mM KCl, 2.5 mM CaCl_2_, 1.2 mM MgCl_2_, 7.7 mM glucose, and 10 mM HEPES (pH 7.2 with NaOH) or in PSS that did not contain CaCl_2_. For fura-2, measurement of intracellular Ca^2+^ levels was performed as described^43^. Changes in intracellular Ca^2+^ levels were calculated using the grynkiewicz equation: [Ca^2+^] = *K*_*d*_ *Q*(*R* − *R*_min_)/(*R*_max_ − *R*). Calibration of the system was performed using a fura-2 calcium imaging calibration kit (Molecular Probes, Eugene, OR), and values were determined to be as follows: *Q* = 23.04; *R*_min_ = 0.31; *R*_max_ = 8.87.

Ca^2+^ levels were also analyzed by confocal microscopy using fluo-4. Samples of NCI-H929 and U266 cells were observed with a Leica TCS SP5 AOBS laser scanning confocal microscope through a 63x/1.4NA Plan Apochromat oil-immersion objective lens (Leica Microsystems CMS GmbH, Germany). Argon 488 laser lines were applied to excite samples, and tunable emissions were used to minimize crosstalk between fluorochromes. Time-lapse (10-sec intervals for 22.5 min) images for each sample were captured with photomultiplier detectors and prepared with LAS AF software version 2.6 (Leica Microsystems). Maximum projection images of individual cells at each time point were analyzed using the Definiens® Developer v2.0 (Definiens AG, Munich, Germany) software suite. Fluo-4-stained cells were segmented by a combination of intensity and size thresholds. Segmented cells were linked through time, and mean intensity data for Fluo-4 at each time point were extracted from the segmentation.

### Cell Death Analysis

After treatment with MTI-101, cells were washed with PBS and incubated with 2 nM TO-PRO-3 iodide (Life Technologies) or FITC Annexin V (BD biosciences) for 45 min. The cells were analyzed for fluorescence with the use of a FACSCalibur (BD Biosciences, San Jose, CA).

### Measurement of cell death by PI staining

U266 and MM1.S (1 × 10^6^ cells/1 ml) cells were incubated with different concentrations of MTI- 101(5, 10 and 20 µM) for 48 hours. Cells were washed 1x in PBS with 1% FBS, resuspended in 500 µl of buffer containing (5 µg/ml of PI) and incubated for 15 min at room temperature in the dark. Samples were analyzed with a BD FACS Calibur flow cytometer (BD Biosciences, San Jose, CA, USA) within 1 h. For each sample, 10 000 events were counted.

### Primary Myeloma Patient Specimens

Patient specimens were from myeloma patients who provided written informed consent through the Moffitt Total Cancer Care® (MCC# 18608) tissue banking protocol per institutional and IRB regulations. Samples were provided to the laboratory as a de-identified sample from the Moffitt Tissue Core. Mononuclear cells were separated from human bone marrow aspirates with the use of Ficoll-Paque PLUS (GE Healthcare, UK). After separation, CD138-positive cells were sorted using MS MACS Separation Columns (Miltenyi Biotec, Germany) and CD138 microbeads (Miltenyi) per manufacturer’s instructions. For flow cytometry assays using Annexin V and FACS analysis to detect dead cells, CD138 cells were seeded at 1 million cells/ml in a 96 well plate (100 ul volume) per patient sample. The cell plating density was consistent across all specimens tested.

#### Stromal cells

Primary MM cells were co-cultured with bone marrow derived stromal cells obtained from patients’ BM aspirates, as previously described^44^. Since this process takes weeks, primary MM cells from fresh biopsies were co-cultured with established stroma from prior patient samples.

### *Ex vivo* assay

The *ex vivo* assay used to quantify chemo sensitivity of primary MM cells was described in detail previously^45^. Briefly, MM cells (CD138+) were seeded in multi-well plates with previously established human-derived stroma and collagen-I to a total volume of 8 µL containing approximately 4,000 MM cells and 1,000 stromal cells. Drugs were added using a robotic plate handler, so that every drug was tested at five concentrations (1:3 serial dilution) and two replicates. Negative controls (supplemented growth media with and without vehicle control, DMSO) were included, as well as positive controls for each drug (cell line MM1.S at highest drug concentration). Plates were placed in a motorized stage microscope (EVOS Auto FL, Life Technologies) equipped with an incubator and maintained at 5% CO_2_ and 37 °C. Each well was imaged every 30 minutes for a total duration of four days.

### Digital image analysis algorithm

We have developed a digital image analysis algorithm previously described^45, 46^ to determine changes in viability of each well longitudinally across the 96 h interval.

### Drug combination and synergy analysis

To study interactions between MTI-101 and bortezomib in patient samples, we have added two drugs diluted serially, so that their concentration ratio remained the same across all wells tested. Thus the concentrations tested were (BTZ/MTI-101): 50 nM/20 µM, 16.7 nM/6.67 µM, 5.6 nM/2.22 µM, 1.9 nM/0.74 µM and 0.6 nM/0.25 µM. To determine if there is synergy between the two drugs, we have plotted the dose-response surface of each drug individually, their actual combination, and the theoretical additive curve (Fig. 6). Here we use the classic definition of synergy, which determines that two drugs are synergistic if their combined effect (percent cells killed) is higher than the combination of their independent effects^47^. For instance, consider that drugs A and B, at a specific concentration and during a certain period of time, kill the fractions *a* and *b* of cells, where *a* < 1 and *b* < 1. Should the combination of both drugs kill a fraction equal to [1 − (1 − *a*) * (1 − *b*)], then A and B are additive. If the fraction killed is higher, the drugs are synergistic; if lower, they are antagonistic.

### Murine 5TGM1 Myeloma Model

Animal studies were conducted using 6- to 8-week-old female C57BL/KaLwRijHsd mice (Harlan) in accordance with the NIH Guide for the Care and Use of Laboratory Animals and protocols were approved by the University of South Florida IACUC committee. 5TGM1 MM cells were injected *i.v*. via tail vein. Establishment of MM tumors in inoculated mice was followed by assaying immunoglobulin G2b (IgG2b) monoclonal paraprotein in sera prepared from whole blood obtained by a submandibular bleed. Mouse IgG2b levels were assayed by an ELISA (Bethyl Laboratories, Montgomery, TX) on days 7, 14, 21, and 28 per manufacturer’s instructions. On day 10 after inoculation, mice began treatment with 10 mg/kg MTI-101 or 0.5 mg/kg bortezomib 3 times/week (Monday, Wednesday, Friday) for 21 days, for nine treatments. All drugs were administered by IP injections. Mice were then examined until euthanization, which occurred when mice displayed hind leg paralysis or tumors grew in excess of 2 cm in diameter. On day 100, all remaining mice were euthanized.

## Electronic supplementary material

Supplementary Information
