# Supplementary material for: MTI-101 treatment inducing activation of Stim1 and TRPC1 expression is a determinant of response in multiple myeloma
Source: Sci Rep. 2017 Jun 2;7:2685. doi: 10.1038/s41598-017-02713-0 (PMC5457439; doi:10.1038/s41598-017-02713-0)
Supplement: Supplementary file 1 — Supplementary Information [file 41598_2017_2713_MOESM1_ESM.doc]

**MTI-101 treatment inducing activation of Stim1 and TRPC1 expression is a determinant of response in multiple myeloma**

**Michael F. Emmons^1,2^, Nagaraju Anreddy ^3^, Javier Cuevas^4^, Kayla Prater^3^, Shengyu Yang^1^, Mark McLaughlin^3^, Ariosto Silva^1^, and Lori A. Hazlehurst^3^**

^1^Tumor Biology Department, Chemical Biology and Molecular Medicine Program, H. Lee Moffitt Cancer Center and Research Institute, Tampa, FL 33612

^2^Modulation Therapeutics, Inc., 3802 Spectrum Boulevard, Suite 124, Tampa, FL 33620

^3^Department of Pharmaceutical Science, University of West Virginia, Morgantown, WV, 26506

^4^Pharmacology and Physiology Department, University of South Florida, Tampa, FL 33620

**Corresponding author:** Lori Hazlehurst, PhD, Department of Basic Pharmaceutical Sciences, University of West Virginia, Morgantown, West Virginia 26506

Email:Lahazlehurst@hsc.wvu.edu

**Supplementary Figure 1 (Original blot of data used in Figure 1C): TRPC1 Expression in H929 AND H929/60 cells.**

**
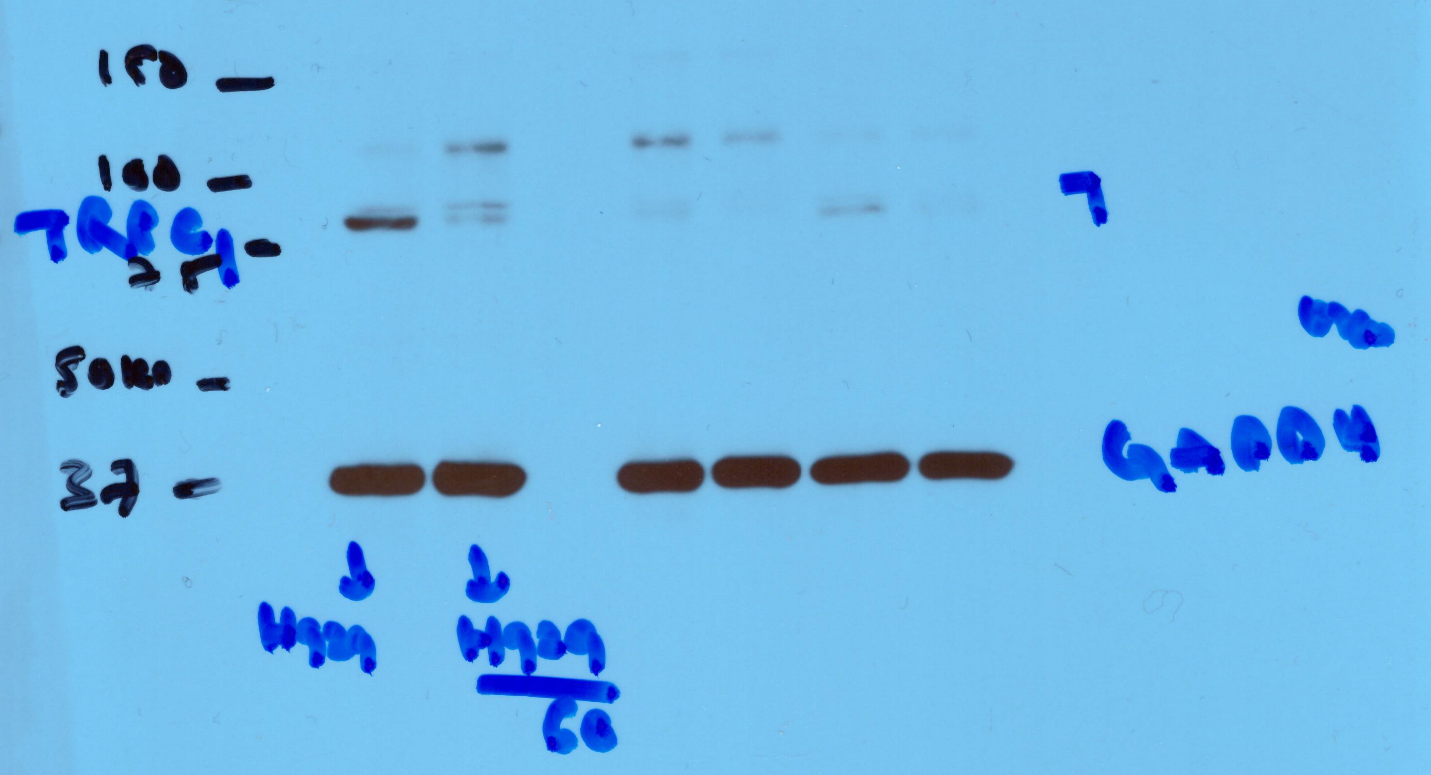
**

TOP Left 1^st^ lane represent TRPC1 expression in H929 Parenteral cell lines, TOP Left 2nd lane represent TRPC1 expression in H929/60 acquired drug resistant cell line (Figure 1C).Bottom bands represents GAPDH levels for respective lanes. TRPC1 1:100 dilution and GAPDH 1:1000 dilution was used.

**Supplementary Figure 2 (Original blot of data used in Figure 1C): PLC-β Expression was measured in in H929 AND H929/60 cells**
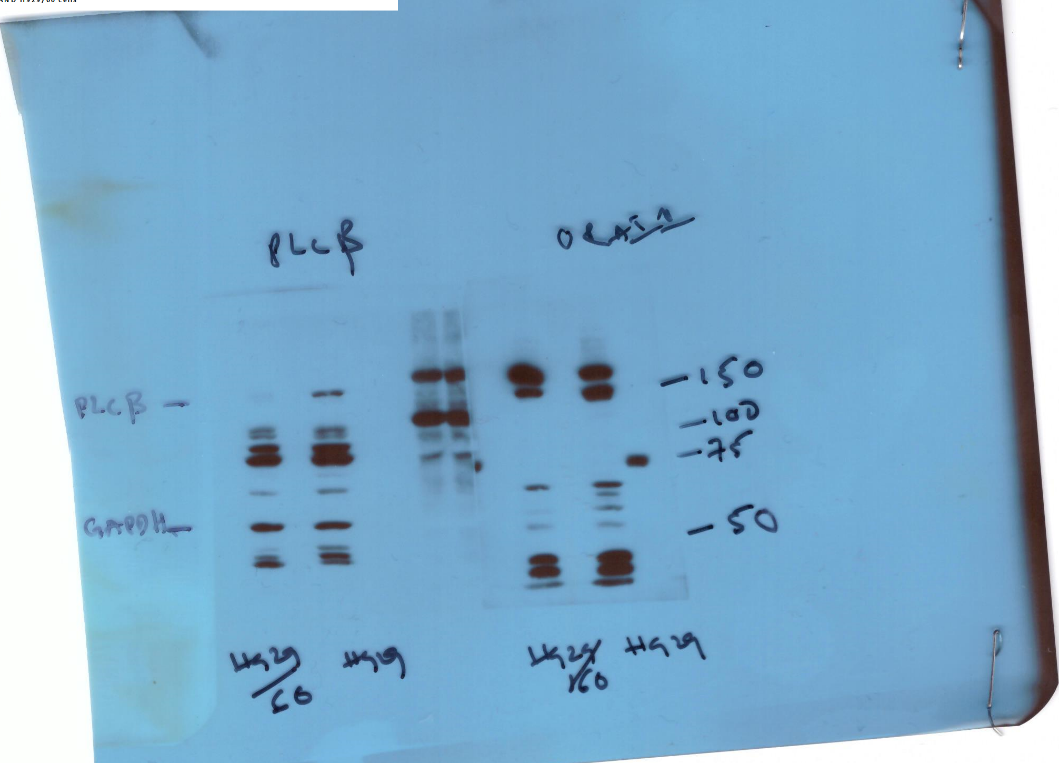


TOP Left 1^st^ lane represent PLC-β expression in H929/60 acquired drug resistant cell line, TOP Left 2nd lane represent PLC-β expression in H929 Parenteral cell line (Figure 1C). Bottom bands represents GAPDH levels for respective lanes. PLC-β 1:100 dilution and GAPDH 1:1000 dilution was used.

**Supplementary Figure 3 (Original blot of data used in Figure 4B). TRPC1 expression after retroviral transfection with TRPC1 SHRNA constructs (A, B AND C) In U266cell lines.**


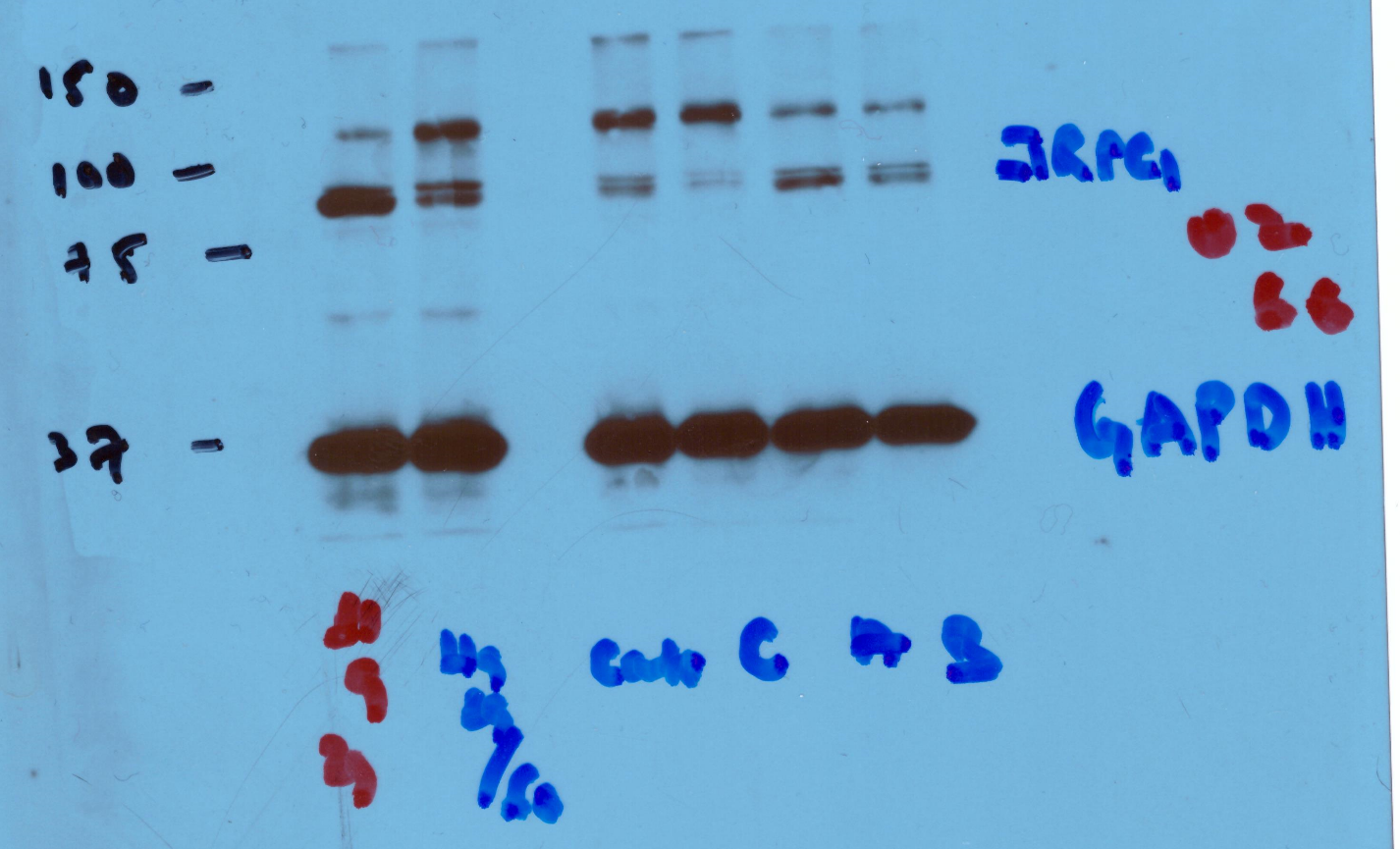


TOP Left 1^st^ lane represent TRPC1 expression in H929 Parenteral cell lines, TOP Left 2nd lane represent TRPC1 expression in H929/60 acquired drug resistant cell line. top Left 3^rd^ lane represent control shRNA construct, top Left 4^th^ lane represent control shRNA construct C( Figure 4B), Top left 5^th^ lane represents TRPC1 Sh RNA construct A and Top left 6 lane represent TRPC1 ShRNA construct B , Bottom bands represents GAPDH levels for respective lanes**.** TRPC1 1:100 dilution and GAPDH 1:1000 dilution was used.

**Supplementary Figure 4 (Original blot of data used in Figure 4C). TRPC1 expression after retroviral transfection with TRPC1 SHRNA constructs (A, B AND C) In MM1.s cell lines**


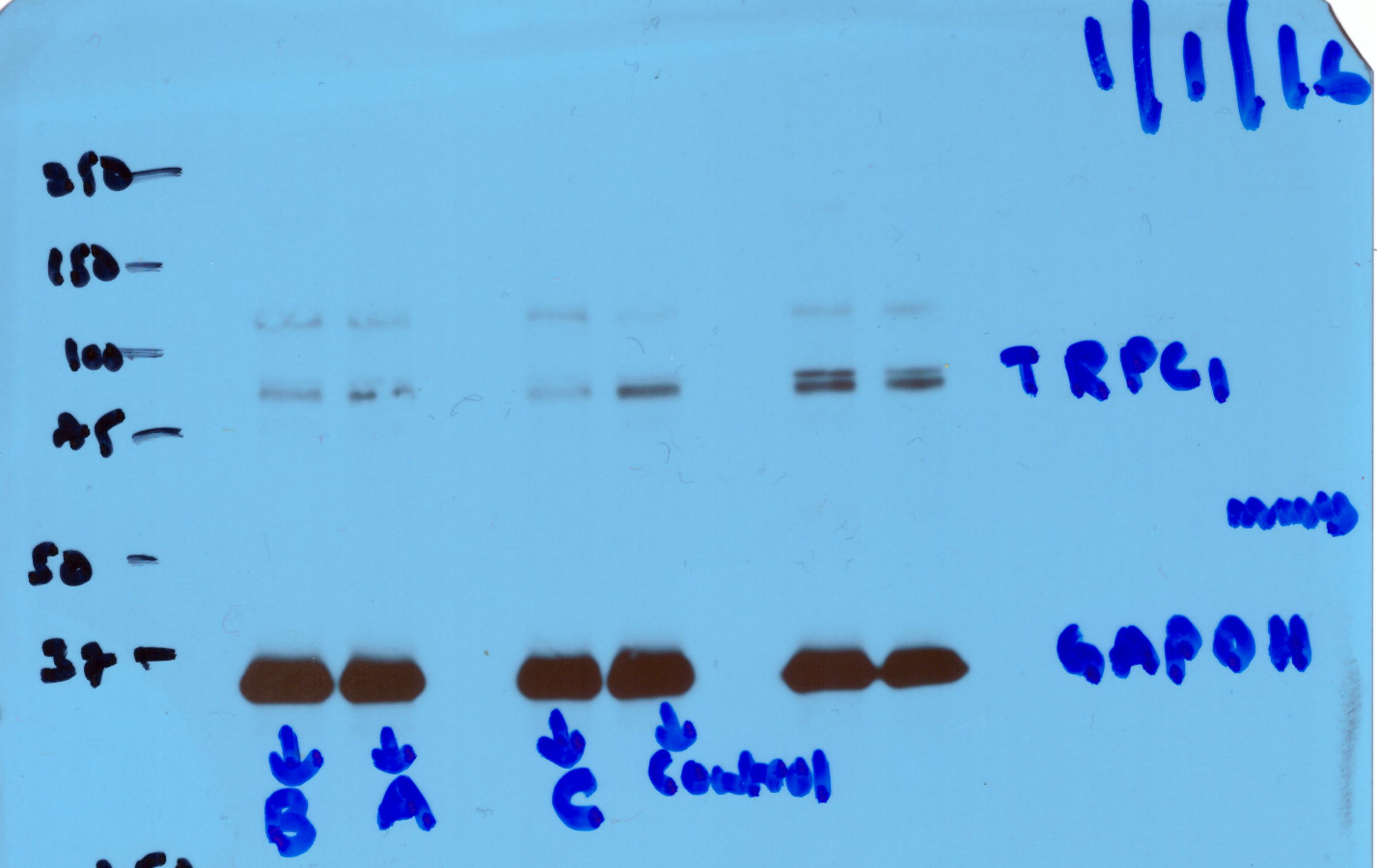


Top membrane represents TRPC1 expression in MM1.S cell lines. TOP Left 1^st^ lane represent TRPC1 Sh RNA construct B, Top left 2^nd^ lane represent TRPC1 ShRNA construct A, top Left 3^rd^ lane represent TRPC1 shRNA construct C and Top left 4^th^ lane represent Control ShRNA. Bottom bands represents GAPDH levels for respective lanes ( GAPDH Bands with lower exposer time was used in manuscript). TRPC1 1:100 dilution and GAPDH 1:1000 dilution was used.
